# Altered frontoparietal activity in acoustic startle priming tasks during reticulospinal tract facilitation: An fNIRS study

**DOI:** 10.3389/fnins.2023.1112046

**Published:** 2023-02-16

**Authors:** Nan Xia, Chang He, Xiupan Wei, Yang-An Li, Weiwei Lou, Minghui Gu, Zejian Chen, Jiang Xu, Yali Liu, Xiaohua Han, Xiaolin Huang

**Affiliations:** ^1^Department of Rehabilitation Medicine, Tongji Hospital, Tongji Medical College, Huazhong University of Science and Technology, Wuhan, Hubei, China; ^2^World Health Organization Collaborating Centre for Training and Research in Rehabilitation, Wuhan, China; ^3^Institute of Medical Equipment Science and Engineering, Huazhong University of Science and Technology, Wuhan, Hubei, China; ^4^State Key Lab of Digital Manufacturing Equipment and Technology, Institute of Rehabilitation and Medical Robotics, Huazhong University of Science and Technology, Wuhan, Hubei, China

**Keywords:** acoustic startle, functional near-infrared spectroscopy, reticulospinal tract, rehabilitation, frontoparietal cortex

## Abstract

**Background:**

Because it is one of the important pathways for promoting motor recovery after cortical injury, the function of the reticulospinal tract (RST) has received increasing attention in recent years. However, the central regulatory mechanism of RST facilitation and reduction of apparent response time is not well understood.

**Objectives:**

To explore the potential role of RST facilitation in the acoustic startle priming (ASP) paradigm and observe the cortical changes induced by ASP reaching tasks.

**Methods:**

Twenty healthy participants were included in this study. The reaching tasks were performed with their left and right hands. Participants were instructed to get ready after the warning cue and complete the reach as soon as they heard the Go cue. Half of the testing trials were set as control trials with an 80-dB Go cue. The other half of the trials had the Go cue replaced with 114-dB white noise to evoke the StartleReact effect, inducing reticulospinal tract facilitation. The response of the bilateral sternocleidomastoid muscle (SCM) and the anterior deltoid was recorded *via* surface electromyography. Startle trials were labeled as exhibiting a positive or negative StartleReact effect, according to whether the SCM was activated early (30–130 ms after the Go cue) or late, respectively. Functional near-infrared spectroscopy was used to synchronously record the oxyhemoglobin and deoxyhemoglobin fluctuations in bilateral motor-related cortical regions. The β values representing cortical responses were estimated *via* the statistical parametric mapping technique and included in the final analyses.

**Results:**

Separate analyses of data from movements of the left or right side revealed significant activation of the right dorsolateral prefrontal cortex during RST facilitation. Moreover, left frontopolar cortex activation was greater in positive startle trials than in control or negative startle trials during left-side movements. Furthermore, decreased activity of the ipsilateral primary motor cortex in positive startle trials during ASP reaching tasks was observed.

**Conclusion:**

The right dorsolateral prefrontal cortex and the frontoparietal network to which it belongs may be the regulatory center for the StartleReact effect and RST facilitation. In addition, the ascending reticular activating system may be involved. The decreased activity of the ipsilateral primary motor cortex suggests enhanced inhibition of the non-moving side during the ASP reaching task. These findings provide further insight into the SE and into RST facilitation.

## Introduction

As part of the extrapyramidal system, the reticulospinal tract (RST) consists of bundles of axons that convey signals from the reticular formation in the brainstem to the spinal cord; it participates in movement control in humans (Baker, 2011). This descending pathway, together with the more well-known corticospinal tract (CST), constitutes the major control system of human voluntary movement (Brownstone and Chopek, 2018). However, compared to our understanding of the function of the CST, that of the RST has rarely been explored in humans. Based on animal research, the RST is thought to control proximal and axial muscles and be primarily responsible for locomotion (Matsuyama and Drew, 2000) and postural adjustment (Schepens and Drew, 2004). However, some recent studies in humans have revealed extensive participation of the RST in muscle contraction and motor control (Smith et al., 2019; Glover and Baker, 2022), and it plays a pivotal role in the remastering of motor control after brain injury (Zaaimi et al., 2012; Jang et al., 2015). The ipsilateral innervation (Boyne et al., 2021; Fisher et al., 2021; Ko et al., 2021) and abundant plasticity of the RST (Glover and Baker, 2020) provide tremendous potential for the recovery of motor function after CST impairment.

However, few methods or approaches have been developed to measure RST function in humans. Currently, most studies on human motor control by the RST utilize either the ipsilateral motor evoked potentials (iMEPs) *via* transcranial magnetic stimulation (Wassermann et al., 1994; Bawa et al., 2004; Maitland and Baker, 2021), the muscle activation latency after acoustic startle (Rangarajan et al., 2022) or a combination of the two (Smith et al., 2019) to deduce its function from target muscles. Through the cortical-reticulospinal pathway, transcranial magnetic stimulation likely indirectly affects the RST and triggers iMEPs of the target muscle (Fisher et al., 2012). The changes in activation latency and amplitude of iMEPs are believed to reflex RST adaptation during strength training and motor recovery from central nervous system injury (Alagona et al., 2001; Atkinson et al., 2022). Typically, iMEPs exhibit higher trigger thresholds and longer latencies than contralateral MEPs in healthy subjects (Bawa et al., 2004). However, in some patients with subcortical defects, a decreased threshold and shorter activation latency were also observed (Alagona et al., 2001). This phenomenon may be explained by the enhanced involvement of the RST in motor control after stroke (Zaaimi et al., 2012; Choudhury et al., 2019).

Acoustic startle stimuli are also widely used to explore the function of the RST in movement. Using a loud sound (>110 dB) as the start signal of a task can evoke the early initiation of prepared movement at an extremely short latency (DeLuca et al., 2022). This phenomenon is called the StartleReact effect (SE) and is recognized as the result of the rapid transmission of motion commands mainly *via* the RST (Carlsen and Maslovat, 2019). Despite not always occurring simultaneously (Leow et al., 2018), early activation of the sternocleidomastoid muscle (SCM) due to the startle reflex is strongly correlated with early initiation of movement in this paradigm (Maslovat et al., 2021). The SCM activation latency in the time window of 30 to 130 ms provides a convenient marker of the SE (Carlsen et al., 2011; van Lith et al., 2018). Therefore, analyses that simultaneously incorporate SCM response time and limb muscle activity enable better dissection of RST function in motor control (Maslovat et al., 2021). With this approach, researchers have revealed increased motor unit discharge (Skarabot et al., 2022), additional muscle contraction (Fernandez-Del-Olmo et al., 2014), a greater range of motion during motor initiation (McInnes et al., 2020), and even better motor (Rahimi and Honeycutt, 2020) or speech output (Swann et al., 2022) in stroke survivors. Given the hardware demands and technical challenges of this approach, using acoustic startle priming (ASP) to assess the characteristics and adaptation of the RST for motor control may be an easier approach.

In some previous studies, auditory stimuli have been found to modulate cortical excitability (Furubayashi et al., 2000; Lofberg et al., 2014), providing input *via* the ascending reticular activating system to the brain cortex (Saper et al., 2005). Furubayashi et al. (2000) were one of the first to examine the effects of acoustic stimuli on the cortex. Their study revealed transient inhibitory effects of sound stimuli on the motor cortex in the resting state. Subsequent studies further confirmed this inhibitory pathway derived from the RST (Fisher et al., 2004; Kuhn et al., 2004). However, the inhibitory effects detected in the resting state were completely reversed during motor preparation. In people highly prepared for action, corticospinal excitability was increased after loud auditory stimuli (Marinovic et al., 2014). A recent study also revealed that inducing the startle effect at the end of movement promotes motor learning and improves task performance (Leow et al., 2021). However, beneficial effects of ASP cannot be attributed to changes in excitability during preparation. The acoustic stimuli did not evoke significant changes in the ipsilateral motor cortex during preparation in a dual-coil transcranial magnetic stimulation (TMS) paradigm (Marinovic et al., 2015). A more recent study based on a combined acoustic startle-TMS paradigm further validated the above findings and disentangled evidence of the cortical effects from the startle effect. The different MEP changes in the M1 at rest and during motor preparation induced by acoustic startle may be indirect and regulated by higher-level centers (Chen et al., 2022). Due to the limitation of TMS paradigms, it may be necessary to use imaging techniques to further verify the existence of this regulatory center.

Although the exact neural mechanism by which loud sounds induce the SE is unclear, it most likely involves some known subcortical and cortical pathways in the brain (Marinovic and Tresilian, 2016; Carlsen and Maslovat, 2019). In mammalian studies, two neural pathways (the cortico-striato-pallido-pontine network and an independent circuit from the central nucleus of the amygdala to the pontine reticular nucleus) have been found to participate in the modulation of prepulse inhibition of the auditory startle reflex (Cano et al., 2021; Zhang et al., 2022). Moreover, activity in the supplementary motor area (SMA), supramarginal gyrus, cingulate cortex, anterior insula and cerebellar lobule was also associated with startle stimuli (Mueller-Pfeiffer et al., 2014). Two hypothetical cortical circuits underlying ASP were proposed by Marinovic and Tresilian (2016) i.e., the startle stimuli may transmit information through the thalamus to the auditory cortex *via* the primary auditory pathway and then through other motor cortices to the primary motor cortex (M1) to form motor commands descending to the spinal cord. Additionally, the stimulus signal can directly reach the pontomedullary reticular formation (PMRF) and then the motor cortex *via* the thalamus to complete motor output. With the activation of these subcortical structures, the ascending reticular activation system (ARAS) is likely to be activated, which arises from the PMRF and has extensive connections with the frontal and parietal cortex, including the sensorimotor network (SMN). In addition, some high-level cortical modulation networks identified from resting-state functional magnetic resonance imaging (fMRI) (Yeo et al., 2011) also have confirmed connectivity with the ARAS (Weng et al., 2017; Wijdicks, 2019). Among them, the triple-network model (Menon, 2011) involving the default mode network (DMN) (Buckner, 2013), the lateral frontoparietal network (FPN) (Uddin et al., 2019), and the salience network (SN) (Menon and Uddin, 2010) has received substantial attention. As part of the central executive network, the FPN is located in the dorsolateral prefrontal cortex (dlPFC) and posterior parietal cortex and is involved in working memory, sustained attention, and problem solving. However, the DMN, which includes regions in similar areas, plays the opposite role. The DMN is active when an individual is not focused on external stimuli. The SN acts as an interface between the two networks; it integrates sensory, emotional and cognitive information to balance external stimuli with internal mental processes (Menon, 2011). These large-scale brain networks cover most of the frontal and parietal cortex and some subcortical regions.

Functional magnetic resonance imaging is an excellent method of measuring cortical activity. However, its limited space precludes large-scale arm and torso movements, as needed for the ASP reaching paradigm that we developed (Xia et al., 2021). Additionally, its magnetic field poses a large challenge to recording equipment. Functional near-infrared spectroscopy (fNIRS) provides another method of observing cortical activation during motor tasks. It is a non-invasive neuroimaging technique that detects changes in the oxygenation of hemoglobin in brain tissue *via* differences in optical absorption (Chen et al., 2020). With advances in data processing, it has been widely used to monitor cortical activation during various cognitive and motor tasks (Chen et al., 2020; Huo et al., 2021). Since fNIRS has less environmental limitations and allows a large range of motion during recording, it is a suitable method for dynamic observation of cortical activity during RST facilitation in the ASP reaching tasks.

In the present study, we aimed to investigate the cortical activation features associated with ASP during movement preparation and further explore the location of potential regulatory centers for the SE and RST facilitation. The bilateral prefrontal cortex, frontal cortex, M1, premotor cortex and SMA were regions of interest. The testing paradigm was consistent with our previous experiment, in which participants were first prompted to enter a state of high movement readiness and subsequently received ASP (Xia et al., 2021). We hypothesized that some motor-related cortices would show different activation in the presence of SE. The results of this study will help to reveal the mechanisms of the ASP-induced SE and RST facilitation. Regulatory centers necessary for RST facilitation can guide future in-depth research.

## Materials and methods

### Participants

A total of 20 volunteers (7 females and 13 males, mean age: 26.26 ± 6.65 years, mean body mass index: 22.80 ± 2.57 kg/m^2^) were invited to participate in this study. All participants were healthy, right-handed, and had good tolerance for sudden 114-dB stimuli. Before participation, all subjects signed informed consent forms. Data from this study were part of a former project that was approved by the Ethics Committee of Tongji Hospital (No. TJ-IRB20210648) and preregistered (No. ChiCTR2100048222).

The sample size was estimated *via* G*Power 3.1 software based on the muscle activation latency of the AD after ASP. According to a recent review (DeLuca et al., 2022), the effect size *d* was set as 0.64, and 17 subjects were needed to detect significance with a paired *t* test with a power of 0.8 and an α level of 0.05. Accounting for a 15% drop-out rate, 20 subjects were needed.

### Experimental procedure

The procedure for this test was exactly the same as that in our previous study (Xia et al., 2021). Participants were asked to sit in front of a blank blackboard in a quiet environment. First, the subject was asked to place their upper limbs next to their trunk and keep their whole bodies relaxed as much as possible. A pallet at 80% of shoulder height was placed on the anterolateral side of the testing limb at a distance of 120% of arm length. Subjects were asked to perform the reaching tasks according to the auditory stimuli from a headphone (Sennheiser HD25-I; Wedemark, Germany). The left and right sides of the subject were tested separately. To maintain sufficient attention during the testing process, three kinds of reaching tasks containing 10 repetitions each were randomly assigned. These tasks included reaching to tap the center of the pallet and reaching to grasp a tennis ball or a coffee takeaway cup with the palm facing inward. Thus, each participant completed a total of 30 trials on their left and right sides.

After 10 consecutive trials, the subjects were allowed to rest and relax for 1 min. Each trial took approximately 23 s. In the first 5 s of the trial, participants were verbally informed of the upcoming task and then heard an 82-dB warning “beep” continuously for 0.5 s to prompt them to be ready. After 2.5–3 s, a 40-ms Go cue was emitted to initiate the aiming task. This sound clip was randomly placed in the aforementioned 500-ms interval to prevent anticipation. Half of the 30 trials were set as control trials, and their Go cues were the same 40-ms “beep” as the warning cue. However, the other 15 trials (startle trials) used a 40-ms 114-dB white noise clip as the Go cue. The order of control or startle trials was also randomized before each test. A 15-s interval was set between every two trials to allow full relaxation. To complete all reach tasks, participants used their left and right hands to perform 30 trials with 80-dB stimuli (control trials) and 114-dB stimuli (startle trials), with 15 trials each. The Psychtoolbox-3 package within MATLAB (2017b, MathWorks, USA) was used to design and implement those tests. A custom-written program was used to simultaneously trigger the markers on the surface electromyography (sEMG) and fNIRS systems as the Go cue was released.

### Surface electromyography and data preprocessing

The Ultium EMG system (Noraxon USA Inc., Scottsdale, AZ, USA) was used to collect the sEMG signals with a sampling rate of 2,000 Hz. Our experimental procedures followed SENIAM recommendations. After the electrodes were connected to the acquisition unit, they were placed on the muscle belly of both (bilateral) SCMs and the anterior deltoid (AD) of the movement side.

Raw sEMG data were processed in MATLAB (2017b, MathWorks, Natick, MA, USA). After data segmentation, the data were bandpass filtered (30–300 Hz) and then notch filtered at 50 Hz. The Teager–Kaiser energy operation was applied to further process the filtered data to achieve higher reliability of muscle onset (Solnik et al., 2010). The threshold method was used to detect the muscle onset time. The threshold was set as the mean + 3 SD of baseline amplitude at a time window of 2,500–500 ms before the Go cue on each trial. The time intervals from the Go cue to muscle onset of SCMs and the AD were recorded as the reaction time and activation latency, respectively, for further analysis.

Trials were first excluded if the AD reaction time did not occur in the time window of 30 to 400 ms after the Go cue. For startle trials, those with a reaction time of either SCM within 30 to 130 ms after the Go cue were marked as a positive startle reaction (SCM^+^) (van Lith et al., 2018). Similarly, those without obvious SCM activation in this interval were marked as a negative startle reaction (SCM^–^). In addition, taking into account the SCM^+^ incidence in the previous study (Xia et al., 2021) and the requirements of fNIRS data analysis, data from subjects with a disproportionately low proportion of SCM^+^ trials (<3/15) were considered invalid and excluded from the analysis.

### Functional near-infrared spectroscopy (fNIRS) and data preprocessing

**FIGURE 1:**
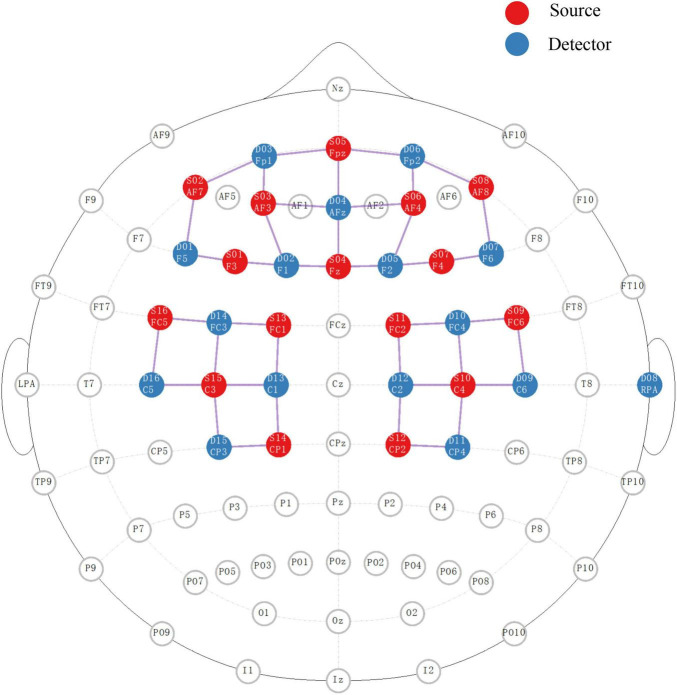
The placement of sources and detectors for fNIRS detection in 10/10 EEG system. The red and blue circles represent the ordered sources and detectors, respectively. The pink line between the two represents the active channel used for this study.

During testing, changes in deoxyhemoglobin (HbR) and oxyhemoglobin (HbO) concentrations were monitored *via* a wearable fNIRS device (NIRSport2, Nirx Medical Technologies LLC, Berlin, Germany). Subjects were asked to rest in a quiet sitting position for more than 1 min before starting the test. Forty valid NIRS channels with 16 dual-wavelength LED sources (760 nm and 850 nm) and 16 detectors were placed to cover the bilateral prefrontal cortex, frontal cortex, M1, premotor cortex and SMA. The distance between the first source and detector was 3.1 cm, and the exact distance of the other channels was automatically calculated by nirsLAB software (version 2017.06, NIRx Medical Technologies, Glen Head, NY, USA). The HbR and HbO concentrations at each location were recorded at a sampling rate of 6.1 Hz. The detailed locations of each source and detector as well as the representative Brodmann area and MNI coordinates of each channel are provided in Figure 1 and Table 1.

**TABLE 1:** Coordinates and cortical areas of the fNIRS channels.

Channel no.	Source-detector	10/10_EEG_system	MNI coordinate			Brodmann area	Specificity (%)
			*X* (mm)	*Y* (mm)	*Z* (mm)		
1	S1-D1	F3-F5	−46	39	26	45–pars triangularis Broca’s area	72.56
2	S1-D2	F3-F1	−31	39	41	9–Dorsolateral prefrontal cortex	66.61
3	S2-D1	AF7-F5	−47	46	6	46–Dorsolateral prefrontal cortex	43.20
4	S2-D3	AF7-Fp1	−33	59	−2	11–Orbitofrontal area	32.71
5	S3-D2	AF3-F1	−23	52	32	9–Dorsolateral prefrontal cortex	48.44
6	S3-D3	AF3-FP1	−24	63	9	10–Frontopolar area	69.63
7	S3-D4	AF3-AFz	−12	62	23	10–Frontopolar area	75.76
8	S4-D2	Fz-F1	−9	41	50	9–Dorsolateral prefrontal cortex	63.16
9	S4-D4	Fz-Afz	2	50	39	9–Dorsolateral prefrontal cortex	61.77
10	S4-D5	Fz-F2	10	41	50	9–Dorsolateral prefrontal cortex	68.93
11	S5-D3	Fpz-Fp1	−12	67	0	10–Frontopolar area	54.50
12	S5-D4	Fpz-AFz	1	64	14	10–Frontopolar area	87.48
13	S5-D6	Fpz-Fp2	13	67	0	10–Frontopolar area	54.46
14	S6-D4	AF4-Afz	13	61	24	10–Frontopolar area	72.47
15	S6-D5	AF4-F2	22	52	33	9–Dorsolateral prefrontal cortex	51.52
16	S6-D6	AF4-Fp2	25	63	9	10–Frontopolar area	68.78
17	S7-D5	F4-F2	30	40	41	9–Dorsolateral prefrontal cortex	68.37
18	S7-D7	F4-F6	48	42	22	46–Dorsolateral prefrontal cortex	82.10
19	S8-D6	AF8-Fp2	34	59	−2	10–Frontopolar area	31.08
20	S8-D7	AF8-F6	48	46	5	46–Dorsolateral prefrontal cortex	43.18
21	S9-D9	FC6-C6	66	−3	24	6–Pre-motor and supplementary motor cortex	66.08
22	S9-D10	FC6-FC4	56	12	33	6–Pre-motor and supplementary motor cortex	40.06
23	S10-D9	C4-C6	62	−20	37	2–primary somatosensory cortex	27.65
24	S10-D10	C4-FC4	52	−4	48	6–Pre-motor and supplementary motor cortex	56.87
25	S10-D11	C4-CP4	53	−35	52	40-Supramarginal gyrus part of Wernicke’s area	50.04
26	S10-D12	C4-C2	42	−21	62	4–Primary motor cortex	36.77
27	S11-D10	FC2-FC4	39	12	54	6–Pre-motor and supplementary motor cortex	38.21
28	S11-D12	FC2-C2	27	−4	68	6–Pre-motor and supplementary motor cortex	82.46
29	S12-D11	CP2-CP4	39	−49	60	40-Supramarginal gyrus part of Wernicke’s area	45.11
30	S12-D12	CP2-C2	28	−36	71	4–Primary motor cortex	31.56
31	S13-D13	FC1-C1	−26	5	68	6–Pre-motor and supplementary motor cortex	81.78
32	S13-D14	FC1-FC3	−38	12	55	6–Pre-motor and supplementary motor cortex	37.52
33	S14-D13	CP1-C1	−27	−36	71	4–Primary Motor Cortex	31.56
34	S14-D15	CP1-CP3	−39	−48	60	40-Supramarginal gyrus part of Wernicke’s area	41.82
35	S15-D13	C3-C1	−42	−20	62	4–Primary motor cortex	34.98
36	S15-D14	C3-FC3	−50	−3	50	6–Pre-motor and supplementary motor cortex	61.71
37	S15-D15	C3-CP3	−52	−34	52	40-Supramarginal gyrus part of Wernicke’s area	43.32
38	S15-D16	C3-C5	−60	−18	37	3–primary somatosensory cortex	23.83
39	S16-D14	FC5-FC3	−55	12	34	44–part of Broca’s area/ 6–Pre-motor and supplementary motor cortex	47.81/35.96
40	S16-D16	FC5-C5	−62	−3	23	43–Subcentral area	47.13

The fNIRS data were processed *via* nirsLAB software (version 2017.06, NIRx Medical Technologies, Glen Head, NY, USA). During data processing, invalid error trials and tests marked during sEMG data processing were excluded from subsequent analysis. The two 1-min resting periods in each test were truncated. Data from 5 s before to 35 s after the Go cue in each trial were retained for analysis. Data from 100 s before the first trial to 50 s after the last trial were also preserved as a baseline reference. The gain setting (Zhang et al., 2018) and coefficient of variation were set at 7 and 15%, respectively, to improve the signal-to-noise ratio. Datasets with over 10 bad channels (out of 40 total channels) were excluded from further analysis. Discontinuities and spike artifacts were removed with a 5-SD threshold. Then, a bandpass filter at 0.01 to 0.09 Hz (Pinti et al., 2018) was used to filter the remaining data. The intensity data were converted into optical density changes and transformed to relative fluctuations of HbO/HbR concentrations by using the modified Beer-Lambert law (Cope and Delpy, 1988). Both the HbO and HbR signals were chosen for subsequent processing. An event time window of 5 s after each warning cue in each trial was set to calculate the hemodynamic response function with HbO/HbR fluctuations based on a generalized linear model. Within-subject statistical parametric mapping (SPM) was used to estimate the β values of each fNIRS channel.

### Statistical analyses

The tasks with the left and right hands were processed and analyzed separately. Due to the similar sEMG responses (Xia et al., 2021), the side differences in the three reaching tasks were neglected in this study. The reaction time of SCM in each kind of trial of each subject was averaged and reported as the mean with standard deviation. The activation latency of AD in the control, SCM^+^ and SCM^–^ trials was averaged for each separate left- or right-side test and reported as the mean with standard deviation. The Shapiro-Wilk test was used to evaluate the normal distribution of variables. After confirming the homogeneity of variance, two-way analyses of variances (ANOVAs) with Bonferroni *post hoc* comparisons were used to identify significant difference in muscle reaction time according to trial type and movement side. Two-way ANOVAs with Bonferroni *post hoc* comparisons were used to detect differences in β values of channels in the same model. However, few significant differences in β values within different trials were found. No difference was detected between the left and right movement sides. A further one-way ANOVA for data from each movement side was also performed. However, the only significant differences among the three kinds of trials were in β values from channel 17 on the HbR response. In addition, the large standard deviations of β values from these channels revealed large interindividual variability. Considering that this study involved a within-subjects design, paired *t* tests with a Bonferroni correction (*P* < 0.05/2 = 0.025) were chosen as an alternative method of detecting within-individual differences in β values between pairs among the 3 kinds of trials. SPSS 24.0 software (SPSS, Chicago, IL, USA) was used for statistical analyses, and the significance was set at *P* < 0.05.

## Results

### Characteristics of valid trials and comparison of muscle activation latency

A total of 40 datasets from the left and right sides of 20 subjects were collected. Among them, 8 datasets were directly excluded from subsequent analysis because there were fewer than 20% of SCM^+^ trials (3/15 trials). Similarly, two other datasets were eliminated due to the number of bad channels in the simultaneously collected fNIRS data (>10/40 channels). Of the remaining 900 trials from 30 datasets, 32 trials were further excluded due to apparent early movement initiation (AD reaction time <30 ms) or delayed initiation (AD reaction time >400 ms). Therefore, data from 30 datasets (15 from the left side to 15 from the right side) consisting of 429 control trials, 166 SCM^+^ trials, and 273 SCM^–^ trials were included in the final analyses. Supplementary Material 1 provides the proportion of SCM^+^ trials in the left- or right-side movements of each subject. The mean positive startle rate (SCM^+^) was approximately 37.95%. The mean SCM response times of the control and SCM^+/–^ trials were 201.62 ± 74.36 ms and 117.31 ± 52.69/141.28 ± 58.95 ms, respectively. Two-way ANOVAs revealed a significant main effect of trial type on SCM response time [*F*(2,825) = 111.76, *P* < 0.001], and the *post hoc* Bonferroni comparisons also revealed significant differences between control and SCM^+^ trials (*P* < 0.001) and between SCM^+^ and SCM^–^ trials (*P* < 0.001). No significant difference was found between control and SCM^–^ trials (*P* > 0.05). Moreover, significant main effects of trial type (SCM^+^, SCM^–^, or control) [*F*(2,867) = 77.88, *P* < 0.001] and movement side [*F*(1,867) = 1.06, *P* = 0.025] on the AD reaction time were detected. Significant differences between each pair of trials were found in the *post hoc* Bonferroni comparisons (*P* < 0.01). The mean AD reaction times of SCM^±^ trials and control trials in the right-side movements were 132.07 ± 39.97/157.84 ± 43.28 ms and 191.65 ± 72.02 ms, respectively. The mean AD reaction times of SCM^+/–^ trials and control trials in the left-side movements were 134.91 ± 49.04/166.01 ± 47.46 ms and 203.76 ± 70.26 ms, respectively. The activation latency of AD in SCM^+^ trials was approximately 60 ms faster than that in control trials.

### Comparisons of β values in fNIRS data among control, SCM^+^, and SCM^–^ trials

Thirty datasets (15 left-side and 15 right-side) from 17 subjects were included in the analyses. The primary outcome was differences in β values of the 40 channels among control, SCM^+^, and SCM^–^ trials.

In the right-side movements, the one-way ANOVA revealed a significant main effect of trial type [*F*(2,42) = 3.57, *P* = 0.037] on the β values of the HbR response in channel 17. The *post hoc* Bonferroni comparisons suggested significantly higher fluctuation in SCM^+^ trials than in SCM^–^ trials (*P* = 0.032). Although this *post hoc* comparison did not reveal a significant difference between SCM and control trials (*P* > 0.05), the difference between the two kinds of trials was revealed *via* a paired *t* test after Bonferroni correction (*t_14_* = −2.858, uncorrected *P* = 0.013). Additionally, the HbR responses in SCM^+^ trials of channel 33 were significantly smaller than those in control trials (*t*_12_ = −2.961, uncorrected *P* = 0.017). However, no differences were found in the other pairwise comparisons after Bonferroni correction (*P* > 0.05). As shown in Table 1 and Figure 1, channel 17 and channel 33 had specificities of 68.37 and 31.56%, respectively, for the right dlPFC and left M1. No differences were found in the β values of HbR responses in other channels or in HbO responses in all channels (*P* > 0.05).

In the left-side movements, paired *t* tests revealed a significant difference in β values from HbR in channel 11 between SCM^+^ trials [(8.32 ± 21.21) × 10^–5^] and control trials [(−0.69 ± 18.48) × 10^–5^] (*t_12_* = −2.976, uncorrected *P* = 0.009). A greater response was also found in SCM^+^ trials than in SCM^–^ trials [(−3.02 ± 22.75) × 10^–5^] (*t_11_* = 2.976, uncorrected *P* = 0.009). No difference was found between the SCM and control trials (*P* > 0.05). Channel 11 had a specificity of 54.50% for the left frontopolar area. In the comparison between SCM^+^ and SCM^–^ trials, paired *t* tests revealed significantly larger and smaller HbO responses of SCM^+^ trials in channels 18 (*t_12_* = −3.132, uncorrected *P* = 0.007) and 30 (*t_12_* = −3.002, uncorrected *P* = 0.010), respectively. However, no differences were found in other pairwise comparisons after Bonferroni correction (*P* > 0.05). The above two channels correspond to the right dlPFC (specificity: 82.10%) and the right M1 (specificity: 31.56%), respectively. The β values of the 3 kinds of trials for every positive channel of HbO/HbR responses are provided in Table 2.

**TABLE 2:** The results of β value comparisons among control, SCM^+^, and SCM^–^ trials in the positive channels.

Channel	Left and right side tasks	HbO/HbR	Trials	β values (×10^–5^)			*Paired-T*	*t*	*Uncorrected P*-values
				Mean	SD	SEM			
			Control	−0.69	18.48	4.62	SCM^+^ vs. Control	−2.98	0.009
11	Left	HbR	SCM^+^	8.32	21.25	5.30	SCM^+^ vs. SCM^–^	2.98	0.009
			SCM^–^	−3.02	70.78	22.75			
			Control	24.03	271.12	70.00	SCM^+^ vs. Control	−2.86	0.013
17	Right	HbR	SCM^+^	156.61	350.21	90.42			
			SCM^–^	−136.49	274.13	70.78			
			Control	1.72	19.15	4.79	SCM^+^ vs. SCM^–^	−3.13	0.007
18	Left	HbO	SCM^+^	−12.43	35.50	8.88			
			SCM^–^	−1.02	32.35	8.09			
			Control	−46.78	920.95	246.14	SCM^+^ vs. SCM^–^	−3.00	0.010
30	Left	HbO	SCM^+^	−280.33	833.95	222.88			
			SCM^–^	343	922.36	246.51			
			Control	−171.48	272.07	90.69	SCM^+^ vs. SCM^–^	−2.96	0.017
33	Right	HbR	SCM^+^	9.28	273.11	91.04			
			SCM^–^	−281.92	489.79	163.26			

SD and SEM represent standard deviation and standard error of mean, respectively.

**FIGURE 2:**
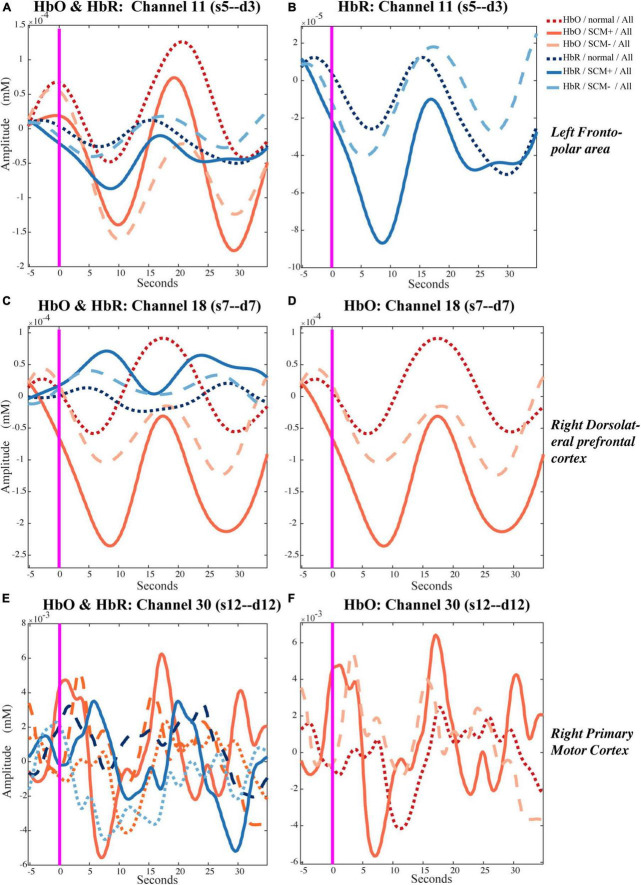
Temporal hemodynamic response of HbO and HbR in control, SCM^+^ and SCM^–^ trials. **(A,B)** Show the hemodynamic responses in HbO/HbR concentrations of channel 11 for control, SCM^+^ and SCM^–^ trials 35 s after the warning cue. In panel **(B)** shows the HbR responses. **(C,D)** Show the hemodynamic HbO/HbR responses and the positive HbO responses in channel 18, respectively. **(E,F)** Show the hemodynamic HbO/HbR responses and the positive HbO responses in channel 30, during the same period.

Each trial took approximately 23 s, so the event-related fluctuations in HbO/HbR concentrations of approximately 0.043 Hz were likely to occur in the relevant cortex. The representative results of the block-averaged hemodynamic response in HbR/HbO concentrations of channel 11 (left frontopolar area), channel 18 (right dlPFC) and channel 30 (left M1) during the left-side tests are provided in Figure 2. Figures 2A, B show the hemodynamic responses in HbO/HbR concentrations of channel 11 for control, SCM^+^ and SCM^–^ trials 35 s after the warning cue. Figure 2B further shows the HbR responses. There was a significantly greater response in SCM^+^ trials than in the other two trials according to paired *t* tests. Figures 2C–F show the hemodynamic HbO/HbR responses and the positive HbO responses, respectively, during the same period.

**FIGURE 3:**
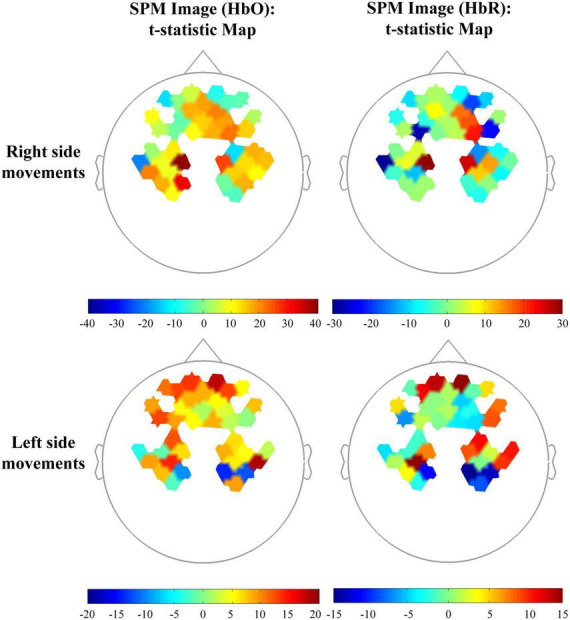
Topographic maps in the comparison among control and SCM^+/–^ trials of one subject. The beta images (HbO and HbR) of SCM^+^ and SCM^–^ trials were set as 1 and –1, respectively for the *t*-statistic map.

Figure 3 displays representative results from one subject and one movement side of statistical parametric mapping analysis of the fNIRS data (HbO and HbR) that were used in pairwise *t* test (1: −1) of brain activation between SCM^+^ and SCM^–^ trials. The differences in activation of the frontal lobe and ipsilateral M1 are shown.

## Discussions

Previous studies investigated the facilitating effects of acoustic startle stimuli on cortical and subcortical areas (Mueller-Pfeiffer et al., 2014; Zhang et al., 2022). However, few studies investigated the activation of the non-motor cortex and potential brain networks. In contrast to commonly used methods of observing brain activation under loud stimuli, we used the ASP motor task to explore the various cortical effects of ASP from movement preparation to movement initiation. As summarized by Marinovic and Tresilian (2016) as the expected moment of motor initiation draws near, preparatory activation of the movement response circuits occurs, and acoustic stimulus-evoked activity can enhance this activation. Sudden stimuli have activating or arousing effects on the sensorimotor system, which could initiate command generation. Moreover, the triggering effect may depend on the amplitude and timing of stimuli. Accordingly, the SE is likely regulated by a higher-level network (Chen et al., 2022). Based on our findings, the possible brain network mechanisms leading to ASP are discussed below.

In the present study, we found that ASP reaching trials with successful RST facilitation (SCM^+^ trials) evoked greater cortical activation in prefrontal areas. This result is consistent with the hypothesized existence of a regulatory center for ASP-induced movements (Chen et al., 2022). Moreover, SCM^+^ trials from both left- and right-side movements exhibited greater activation in the right dlPFC than control or SCM^–^ trials. Furthermore, consistent with previous findings that there was no obvious activation of the M1 after acoustic startle stimuli (Mueller-Pfeiffer et al., 2014; Chen et al., 2022), we found a potential inhibitory effect of the startle on the ipsilateral M1 during RST facilitation. The M1 ipsilateral to the movement side showed a smaller HbR/HbO response during the ASP reaching trials.

### Additional ASP-induced activation of prefrontal areas

Additional activation in the left frontopolar area (Brodmann area 10) was found during the left-side ASP reaching tasks in SCM^+^ trials. The anterior portion of the prefrontal cortex in the human brain is involved in memory recall, decision-making, and various executive functions (Ramnani and Owen, 2004; Koechlin and Hyafil, 2007). Previous studies have noted that anterior prefrontal activity occurs in motor preparation for a cued movement (Sahyoun et al., 2004). This cortical activity, which represents the level of attentional focus and movement readiness, is obviously closely related to the occurrence of the SE and the reduced reaction time during RST facilitation (Carlsen et al., 2012; Leow et al., 2018). These areas typically play an inhibitory role in motor execution (Brass et al., 2001).

Moreover, the additional activation of the right dlPFC was more prominent in SCM^+^ trials of both left and right ASP reaching tasks. This result highlights the important role of the right dlPFC in RST facilitation. It is well known that the dlPFC plays a key role in motor planning, organization, and regulation (Kaplan et al., 2016). Additionally, the FPN to which the dlPFC belongs serves as a flexible hub to rapidly instantiate task states through interactions with other control and processing networks (Marek and Dosenbach, 2018). This network is activated during motor sequence tasks and contributes to motor learning (Maruyama et al., 2021). Simultaneous activation of the FPN and M1 during motor preparation was also found (Maruyama et al., 2021). In addition, the FPN was also found to receive projections from the ARAS, which is primarily responsible for consciousness (Jang et al., 2021). The connection strength between the FPN and ARAS revealed a clear positive correlation with the state of consciousness. Patients in a vegetative state showed a substantial reduction in the connectivity strength between the PMRF and the frontal cortex (Jang et al., 2021). More precisely, in the ARAS, one dopamine signaling pathway arising from the ventral tegmental area has been proven to play a facilitative role in the frontal cortex (Brown et al., 2011). Additionally, supported by the ARAS, the signal from the PMRF can also reach the motor cortex *via* the thalamus and cause excitability of the motor cortex (Brown et al., 2011; Marinovic and Tresilian, 2016). Thus, after activating the cochlear nucleus by loud acoustic stimuli, an upward signal will reach the PMRF and then activate the FPN and motor cortex.

In addition, the right dlPFC may also be involved in threat-induced anxiety. Greater activity in the right dlPFC was found in subjects who classified themselves as behaviorally inhibited (Shackman et al., 2009). In our experiment, a 114-dB acoustic stimulus was used for the Go cue of the prepared reaching task. In addition to inducing the SE, it also induced a sense of threat and anxiety.

Dissociable contributions of the right and left dlPFC to different task demands have been investigated previously (Kaller et al., 2011), and the right dlPFC may be more involved in the planning of simple tasks such as ASP reaching. Moreover, some evidence also indicates lateralization of the PFN. The left PFN may be more strongly related to language function (Smith et al., 2009) rather than movement. The right dlPFC is also better at reactive inhibition (van Belle et al., 2014). Thus, activation of the right dlPFC did not appear to differ between left and right ASP tasks.

### Enhanced inhibitory effect of ASP on the ipsilateral M1

Typically, activation of the M1 is unlikely to occur prior to movement onset. The ipsilateral motor cortex (resting side) is in an inhibited state during motor preparation and execution (Leocani et al., 2000). In a study based on the dual TMS paradigm, Marinovic et al. (2015) found that MEPs on the resting side were significantly facilitated only after movement initiation of the acting side. There is robust evidence that the ipsilateral M1 is significantly suppressed during this period. This inhibition of M1 activity ipsilateral to the movement side may involve cortico-cortical and subcortico-cortical circuits.

The interhemispheric inhibition of the two sides of the M1 or from the motor-related cortex in the contralateral hemisphere has been well explored in previous studies (Ni et al., 2009; Perez and Cohen, 2009). It is generally believed that the involvement of transcallosal glutamatergic pathways links the pyramidal tract with GABAergic interneurons (Reis et al., 2008). It has also been noted that dysgenesis of the corpus callosum significantly affects the function of the FPN (Hearne et al., 2019). Therefore, it can be inferred that there is a potential connection between the FPN and the corpus callosum. Since the corpus callosum plays a key role in interhemispheric inhibition, the FPN may be indirectly involved in this inhibitory regulation of the contralateral motor cortex. However, the exact mechanism remains unclear.

In addition, the FPN may play a facilitating role in the motor cortex responsible for the current movement. In some sequential motor-learning tasks, the FPN and M1 contralateral to the movement side exhibit simultaneous activation during the preparation phase (Maruyama et al., 2021). This is explained as internal reproduction and learning during the motor preparation phase. After the hypothetical motor commands were generated in one side of the M1, a well-timed activation signal from the PMRF-ARAS triggered its early release, which was quickly output to the periphery *via* the RST descending pathway (Marinovic and Tresilian, 2016; Carlsen and Maslovat, 2019). This activation *via* the ARAS is likely to induce a signal that ascends bilaterally but may be ineffective for the contralateral M1, where no motor commands are generated.

The inhibition of the excitability of the ipsilateral M1 in this study may be attributable to our modification of the testing paradigm. In those TMS tests (Marinovic et al., 2015; Chen et al., 2019, 2022), all trials with loud startle stimuli were involved, but our study suggested that approximately half of the startle stimuli did not induce the SE. The occurrence of SE has been confirmed to be highly related to the level of preparation (Leow et al., 2018). Therefore, the absence of a clear definition of successful induction of the SE may dilute the positive trials and thus cause underestimation of this effect in the analyses. In addition, this cortical activity might be task specific. The finger or arm movements used in their tests may evoke lower cortical activation when compared with the reaching tasks, which involve substantial trunk and proximal joint movements. Since the measurement channel for the M1 had low specificity in this study, further confirmation is still needed. Furthermore, as suggested by other researchers (Carlsen et al., 2003; Carlsen and Maslovat, 2019; Maslovat et al., 2021), using SCM^+^ as a marker of successful induction of the SE should be considered in future studies.

In addition, the SN, which is in the same triple-network model as the FPN, may also influence activation. It is mainly responsible for detecting salient events and initiating appropriate control signals to other cortices or networks (Menon and Uddin, 2010; Menon, 2011). In the present study, it seemed to produce a stronger facilitation effect than control or SCM^–^ trials on the right FPN during RST facilitation. Since the regions of interest designated in the present study did not include the anterior insula and anterior cingulate cortex, regions in the SN, further verification is needed.

### Interpretation of the negative fluctuation in HbO/HbR signals

From previous studies, we know that the frontal cortex plays an important role in premotor information processing and motor task preparation (Sahyoun et al., 2004; Koechlin and Hyafil, 2007). Activation of the frontal cortex occurs before task execution and decreases with the advent of movement (Suzuki et al., 2008). Moreover, our ASP paradigm clearly required a long preparation period for the task, which was not limited to the time period starting with the warning cue but also the period after receiving the task information. During these tasks, the subjects need to get into a highly prepared state before movement execution, which is closely related to the delay of motion initiation (Leow et al., 2018). That is, a spike in HbO/HbR changes for the block-averaged figures generated in this study occurred in or near the baseline (time window −5∼0 s), indicating high activation of the frontal cortex as well as increased blood flow. As a result, HbO changes across the channel exhibited task-related negative fluctuations, as Suzuki et al. (2008) reported. The lagged negative fluctuations in HbO signals may reflect different activation levels in the frontal cortex among trials during the motor preparation period. Therefore, a larger negative fluctuation after baseline may represent more cortical activation during the period of motor preparation. We interpreted the HbR fluctuation in channel 11, which had the same negative trend as HbO, as a manifestation of blood flow fluctuations. The effect of blood flow may be greater than that of pure HbR fluctuations. Since the baseline from which the graph (Figure 2) was generated was not highly consistent with the β value calculation (resting state before and after the test), the results may have included some variability.

### Limitations

This study also has some limitations. First, not all signal noise caused by vascular or blood pressure (Scholkmann et al., 2022) was removed, but the current filter parameter settings cover most physiological signals. Moreover, we performed within-subject comparisons that may be able to circumvent the decreased test power due to interindividual differences. In addition, there were significant individual differences in HbO/HbR fluctuations; thus, the contrast map of brain activation in a single subject may not be a good way to explain our results. The subject whose brain activation signatures best represented our findings was selected and presented. Taking into account the reliability and presentability of the findings, we retained the results before correction for multiple comparisons in the SPM figures.

Furthermore, fNIRS itself has some inherent limitations. First, the low sampling rate makes the final data unable to accurately reflect the entire process and temporal order of cortical HbO/HbR fluctuations. Cortical activity, motor initiation, and feedback adjustment in the ASP reaching tasks are completed within tens of milliseconds. EEG, which has a higher sampling rate may be a better method to investigate the coherence between cortical signals and muscular performance. Second, in setting the fNIRS data collection channels, we failed to focus on the ipsilateral motor areas. The setting channels displayed low specificity (31.56%) for the M1. An improved paradigm with short-separation channels may bring better results (Yucel et al., 2021). Moreover, we initially explored the feasibility of using fNIRS to analyze ASP in healthy people; further exploration is needed to determine its application in patients with brain injury. Some preliminary research has suggested that RST facilitation under startle stimuli in patients with cortical injury may be more pronounced and differ from that in healthy individuals (DeLuca et al., 2022; Swann et al., 2022). More ASP movement paradigms with greater sample sizes are needed.

## Conclusion

In summary, this study found that activation of prefrontal regions was significantly associated with the SE and RST facilitation during ASP reaching tasks. Additional activation was most pronounced in the right dlPFC in SCM^+^ trials during this process. Moreover, enhanced inhibition of the ipsilateral M1 was also observed. The above findings suggest a PMRF-ARAS-FPN modulation system for motor output during RST facilitation. The right dlPFC may play an important role in this process. These results can inform future studies on RST facilitation from the perspective of brain networks and support the development of neuromodulation technology to support RST function *via* non-invasive stimulation. Such novel rehabilitation strategies may provide stroke survivors with additional benefits.

## Data availability statement

The raw data supporting the conclusions of this article will be made available by the authors, without undue reservation.

## Ethics statement

The studies involving human participants were reviewed and approved by the Ethics Committee of Tongji Hospital. The patients/participants provided their written informed consent to participate in this study.

## Author contributions

XH, XH, and NX designed this research. NX, CH, Y-AL, MG, ZC, XW, JX, and WL participated into the participants recruitment, research implementation, and data collection. NX and CH did the data analysis and wrote the draft. YL participated the modification of this manuscript. All authors had full access to the data and have reviewed this research and approved the submitted version.
