# Supplementary material for: Altered frontoparietal activity in acoustic startle priming tasks during reticulospinal tract facilitation: An fNIRS study
Source: Front Neurosci. 2023 Feb 16;17:1112046. doi: 10.3389/fnins.2023.1112046 (PMC9978531; doi:10.3389/fnins.2023.1112046)
Supplement: Supplementary file 1 [file Table_1.DOCX]

**Supplementary material 1. proportion of valid control/SCM^+^/SCM^-^ trails for each subject**

| **Subject No.** | **Testing side (Left/Right)** | **Gender (M/F)** | **Number of valid control trials** | **Number of valid SCM^+^ trials** | **Number of valid SCM^-^ trials** | **Proportion of SCM^+^**  **(%)** |
| --- | --- | --- | --- | --- | --- | --- |
| 1 | R | F | 14 | 3 | 12 | 20 |
| 2 | R | M | 15 | 4 | 11 | 26.67 |
| 3 | R | M | 15 | 4 | 10 | 28.57 |
| 3 | L | M | 15 | 4 | 11 | 26.67 |
| 4 | R | F | 15 | 5 | 8 | 38.46 |
| 4 | L | F | 14 | 5 | 10 | 33.33 |
| 5 | R | M | 14 | 3 | 12 | 20 |
| 5 | L | M | 15 | 4 | 11 | 26.67 |
| 6 | R | M | 15 | 7 | 8 | 46.67 |
| 6 | L | M | 15 | 4 | 11 | 26.67 |
| 7 | R | F | 13 | 7 | 8 | 46.67 |
| 7 | L | F | 15 | 3 | 12 | 20.00 |
| 8 | R | F | 14 | 12 | 3 | 80.00 |
| 8 | L | F | 15 | 6 | 8 | 42.86 |
| 9 | R | M | 15 | 3 | 12 | 20.00 |
| 9 | L | M | 14 | 5 | 10 | 33.33 |
| 10 | R | M | 15 | 12 | 3 | 80.00 |
| 10 | L | M | 15 | 7 | 8 | 46.67 |
| 11 | R | F | 15 | 4 | 11 | 26.67 |
| 11 | L | F | 15 | 4 | 10 | 28.57 |
| 12 | L | F | 13 | 3 | 12 | 20.00 |
| 13 | R | M | 13 | 5 | 10 | 33.33 |
| 13 | L | M | 12 | 3 | 10 | 23.08 |
| 14 | R | M | 15 | 9 | 4 | 69.23 |
| 14 | L | M | 14 | 9 | 6 | 60.00 |
| 15 | R | M | 15 | 11 | 3 | 78.57 |
| 15 | L | M | 14 | 6 | 9 | 40.00 |
| 16 | R | F | 13 | 5 | 9 | 35.71 |
| 16 | L | F | 13 | 5 | 10 | 33.33 |
| 17 | L | M | 14 | 4 | 11 | 26.67 |
